# The Role of Butyrate in People with Metabolic Dysfunction-Associated Steatotic Liver Disease and Related Metabolic Comorbidities: A Systematic Review

**DOI:** 10.1007/s13679-026-00694-8

**Published:** 2026-03-04

**Authors:** Alicia González-González, Virginia Soria-Utrilla, María Isabel Fontalba-Romero, María Ángeles Núñez-Sánchez, Antonio Adarve-Castro, María Isabel Queipo-Ortuño, Bruno Ramos-Molina, José Ignacio Martínez-Montoro, José Carlos Fernández-García

**Affiliations:** 1 Department of Endocrinology and Nutrition, Instituto de Investigación Biomédica de Málaga y Plataforma en Nanomedicina (IBIMA-Plataforma BIONAND), Hospital Regional Universitario de Málaga, Málaga, Spain; 2 Department of Nursing and Podiatry, Faculty of Health Sciences, University of Malaga, Malaga, Spain; 3 Obesity, Diabetes and Metabolism Research Group, Biomedical Research Institute of Murcia (IMIB), Murcia, Spain; 4 Department of Radiology, Hospital Universitario Virgen de la Victoria, Málaga, Spain; 5 Department of Surgical Specialties, Biochemical and Immunology, Faculty of Medicine, University of Málaga, Málaga, Spain; 6 Medical Oncology Clinical Management Unit, Virgen de la Victoria University Hospital, Málaga Biomedical Research Institute (IBIMA)- CIMES-UMA, Malaga, Spain; 7 Department of Endocrinology and Nutrition, Instituto de Investigación Biomédica de Málaga y Plataforma en Nanomedicina (IBIMA-Plataforma BIONAND), Hospital Universitario Virgen de la Victoria, Málaga, Spain; 8 Centro de Investigación Biomédica en Red de la Fisiopatología de la Obesidad y Nutrición (CIBEROBN), Instituto de Salud Carlos III, Madrid, Spain; 9 Department of Medicine and Dermatology, Faculty of Medicine, University of Malaga, Malaga, Spain; 10 Centro de Investigación Biomédica en Red de Diabetes y Enfermedades Metabólicas Asociadas (CIBERDEM), Instituto de Salud Carlos III, Madrid, Spain

**Keywords:** Butyrate, Gut microbiota, Metabolic comorbidities, Metabolic dysfunction-associated steatotic liver disease (MASLD), Short-chain fatty acids (SCFAs)

## Abstract

**Background:**

Metabolic dysfunction–associated steatotic liver disease (MASLD) is the most prevalent chronic liver disease worldwide and is strongly linked to obesity, type 2 diabetes, and cardiovascular disease. Growing evidence highlights the role of the gut–liver axis, particularly microbial metabolites such as the short-chain fatty acid (SCFA) butyrate, in MASLD pathophysiology. However, clinical data on butyrate levels and the abundance of butyrate-producing bacteria in MASLD patients remain inconsistent.

**Objectives:**

To systematically synthesize human evidence evaluating the associations between butyrate levels and butyrate-producing gut bacteria with MASLD presence and severity, as well as related metabolic comorbidities.

**Methods:**

A systematic search was conducted in PubMed and Embase from inception to April 7, 2025, following PRISMA 2020 guidelines (PROSPERO registration CRD420251162439). Eligible studies included observational human research assessing fecal or plasma SCFA concentrations and/or the abundance of butyrate-producing taxa in adults with MASLD and related metabolic disorders. Study quality was appraised using the Newcastle–Ottawa Scale, and results were narratively synthesized due to heterogeneity across methods and outcomes.

**Results:**

From 233 records, seven studies met inclusion criteria (2020–2025; *n* = 1,185). Most were cross-sectional or case–control designs of moderate to high quality (NOS 6–8/9). Individuals with MASLD generally exhibited lower fecal or serum butyrate concentrations and reduced abundance of *Faecalibacterium prausnitzii*, *Eubacterium*, and other butyrate-producing bacteria versus controls. These alterations were associated with hepatic steatosis, fibrosis, inflammation, and adverse metabolic profiles - higher BMI, insulin resistance, and dyslipidemia. Geographic and sex-related differences were also reported.

**Conclusions:**

This systematic review suggests that reduced butyrate availability and alterations in butyrate-producing gut taxa are associated with MASLD presence and severity and with adverse metabolic traits. However, substantial methodological heterogeneity and the observational design of available studies preclude causal inference. Larger, well-phenotyped, multicentre studies using standardized SCFA quantification, dietary and medication ascertainment, and functional microbiome profiling are needed to validate these findings and clarify their diagnostic and therapeutic implications.

**Graphical Abstract:**

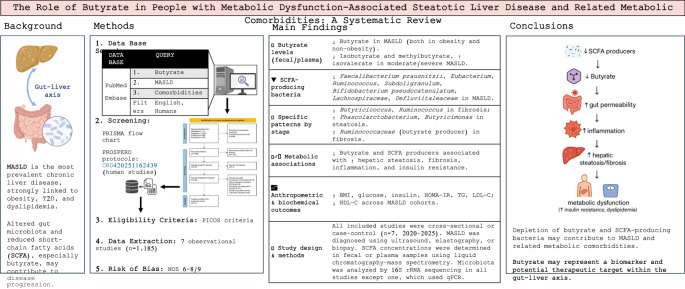

**Supplementary Information:**

The online version contains supplementary material available at 10.1007/s13679-026-00694-8.

## Introduction

Metabolic dysfunction-associated steatotic liver disease (MASLD), formerly known as non-alcoholic fatty liver disease (NAFLD) and/or metabolic dysfunction-associated fatty liver disease (MAFLD), is the most prevalent chronic liver condition worldwide, affecting over 30% of adults and up to 60–70% of individuals with obesity or type 2 diabetes (T2D) [1–4].

MASLD encompasses a spectrum of liver abnormalities ranging from simple steatosis to metabolic dysfunction-associated steatohepatitis (MASH), which is mainly characterized by lobular inflammation, hepatocyte ballooning, and steatosis. MASH can progress to fibrosis, cirrhosis, and hepatocellular carcinoma, and it is strongly related to cardiovascular events. Its progression reflects not only a liver-specific injury but also systemic metabolic dysfunction [5, 6]. Cardiovascular disease remains the leading cause of mortality in patients with MASLD, while comorbidities such as insulin resistance/T2D, dyslipidemia, and visceral adiposity aggravate both hepatic and extrahepatic outcomes [6, 7].

Despite its high prevalence and clinical impact, effective pharmacological treatments for MASLD remain limited. In line with this, resmetirom and semaglutide have recently been approved by the U.S. Food and Drug Administration (FDA) for the treatment of MASH with moderate to advanced fibrosis (F2-F3), representing a major advancement in this field, but further research is still needed to develop effective strategies for this complex disease [8, 9].

In this context, the gut–liver axis has emerged as a potential therapeutic target for MASLD [10, 11]. Dysbiosis is a frequent finding in this condition, commonly characterized by reduced microbial diversity and depletion of beneficial bacteria such as *Faecalibacterium prausnitzii* and *Roseburia* spp., key producers of short-chain fatty acids (SCFAs), particularly butyrate [10, 12, 13]. These alterations may be associated with increased gut permeability and systemic inflammation, processes that have been implicated in liver injury [11, 14].

Butyrate, produced by microbial fermentation of dietary fibers, is the main energy source for colonocytes and plays an essential role in epithelial integrity, immune modulation, and intestinal homeostasis [14, 15]. In animal models, butyrate has been shown to exert systemic effects including improved insulin sensitivity, regulation of hepatic lipid metabolism, and anti-inflammatory actions [10, 16, 17]. Indeed, preclinical evidence suggests that oral butyrate supplementation or dietary strategies to enhance butyrate-producing bacteria may attenuate steatosis, liver inflammation, and fibrosis [15, 17].

Taken together, clarifying the associations between butyrate, butyrate-producing bacteria, MASLD presence and severity may provide insight into its pathophysiology and help identify potential biomarkers or therapeutic targets [18–20]. Hence, in this systematic review we aim to synthesize current clinical evidence on the relationship between butyrate levels, butyrate-producing gut bacteria, MASLD and its metabolic comorbidities.

## Materials and Methods

### Registration of Review Protocol

This systematic review was conducted and reported according to the Preferred Reporting Items for Systematic Reviews and Meta-Analyses (PRISMA) Statement and the PRISMA 2020 checklist [21]. The research protocol was registered at the PROSPERO database (registration number: CRD420251162439).

### Search Strategy

We conducted a comprehensive literature search in the PubMed and Embase electronic databases from inception to April 7, 2025. The search strategy combined controlled vocabulary and free-text terms encompassing three main concepts: butyrate and short-chain fatty acids (SCFAs), steatotic liver disease, and metabolic comorbidities. Specifically, the search string included the term “butyr*” to capture all potential variations reported in the literature (e.g., “butyrate”, “butyric acid”), along with related terms such as “short-chain fatty acids,” “NaB,” “SoB,” and “SCFA”. These were combined with terms describing liver diseases (“non-alcoholic fatty liver disease,” “NAFLD,” “MAFLD,” “MASLD,” “steatosis,” “NASH,” “MASH”, “liver fibrosis,” “cirrhosis”) and metabolic conditions (“type 1 diabetes,” “type 2 diabetes,” “metabolic syndrome,” “insulin resistance,” “obesity,” “cardiovascular disease,” “cardiovascular risk”).

No filters restricting study design (e.g., clinical trials) were applied, in order to ensure comprehensive coverage of observational evidence. The complete search strategy for each database is presented in Table 1.

**Table 1:** Search strategy

PubMed	1	(Butyr* OR “butanoic acid” OR NaB OR SoB OR SCFA OR “short-chain fatty acid”)
	2	(“non-alcoholic fatty liver disease” OR NAFLD OR “metabolic-associated fatty liver disease” OR MAFLD OR “metabolic dysfunction-associated steatotic liver disease” OR MASLD OR “steatosis” OR “steatohepatitis” OR NASH OR MASH OR “liver fibrosis” OR “cirrhosis” OR “fatty liver”)
	3	(“type 1 diabetes” OR T1D OR T1DM OR “Type 2 Diabetes” OR T2D OR T2DM OR “metabolic syndrome” OR “insulin resistance” OR obesity OR “cardiovascular disease” OR “cardiovascular risk” OR “cardiovascular event”)
	4	#1 AND #2 AND #3 NOT Review [Publication Type]
	5	Filters: English, Humans
Embase	1	(butyr* OR ‘butanoic acid’ OR nab OR sob OR scfa OR ‘short chain fatty acid’)
	2	(‘non-alcoholic fatty liver disease’ OR nafld OR ‘metabolic associated fatty liver disease’ OR mafld OR ‘metabolic dysfunction associated steatotic liver disease’ OR masld OR ‘steatosis’ OR ‘steatohepatitis’ OR nash OR mash OR ‘liver fibrosis’ OR ‘cirrhosis’ OR ‘fatty liver’)
	3	(‘type 1 diabetes’ OR t1d OR t1dm OR ‘type 2 diabetes’ OR t2d OR t2dm OR ‘metabolic syndrome’ OR ‘insulin resistance’ OR obesity OR ‘cardiovascular disease’ OR ‘cardiovascular risk’ OR ‘cardiovascular event’)
	4	#1 AND #2 AND #3
	5	#4 AND (‘article’/it)
	6	#5 AND [english]/lim AND [humans]/lim

The search was restricted to studies involving human participants and published in English. Review articles were excluded. Two reviewers (VS-U and AG-G) independently screened titles and abstracts and assessed the full texts of potentially relevant studies. Any disagreements were resolved through discussion and consensus. In cases where consensus could not be reached, a third reviewer (JCF-G) was consulted to provide a final decision.

### Eligibility Criteria

Publications were included for screening if they contained the keywords described in the search strategy and met the PICOS criteria, which are summarized in Table 1. Eligible studies had to meet the following inclusion criteria: (a) original research articles published in peer-reviewed journals; (b) human studies; (c) adult participants diagnosed with MASLD and presenting metabolic comorbidities associated with MASLD, such as obesity, diabetes, insulin resistance, metabolic syndrome, or high cardiovascular risk; (d) studies that evaluated butyrate or other SCFA levels (either fecal or plasma), or the abundance of butyrate-producing microbiota; and (e) studies published in English. Included studies also had to report at least one MASLD-related outcome (e.g., hepatic parameters, liver fat assessed by imaging techniques or biopsy, or liver fibrosis assessed by transient elastography or biopsy), and/or metabolic outcomes (e.g., glucose metabolism, lipid profile, body mass index [BMI]).

The exclusion criteria were: [1] narrative reviews, systematic reviews, meta-analyses, editorials, conference abstracts, case reports, protocols, commentaries, and other non-original papers; [2] *in vitro* or animal studies; [3] studies not measuring or reporting butyrate/SCFA levels or associated microbiota; [4] studies not reporting metabolic or hepatic outcomes; and [5] studies with unclear data or insufficient methodological quality.

Given that this systematic review aimed to synthesize the current evidence on the associations between endogenous production of butyrate (or other SCFAs) and MASLD, clinical trials and interventional studies were also excluded.

MASLD definitions were those applied in each individual study. No limitations were applied regarding year of publication. The PICOS (Participants, Intervention, Comparator, Outcomes, Study design) framework and the criteria are summarized in Table 2.

**Table 2:** Inclusion and exclusion criteria

Parameter	Inclusion Criteria	Exclusion Criteria
Population (P)	Adults with MASLD and associated metabolic comorbidities (obesity, diabetes, insulin resistance, metabolic syndrome, or cardiovascular risk)	Studies conducted in animals or *in vitro*; studies in pediatric populations
Interventions/Exposition (I)	Presence of MASLD (any grade)	Studies not assessing MASLD
Comparison (C)	Healthy controls without MASLD (or without metabolic comorbidities)	Studies lacking a comparator group or using inappropriate comparison groups for MASLD status
Outcomes (O)	Differences in SCFA levels or SCFA-producing microbiota, and its relationship with MASLD-related parameters (e.g., liver enzymes, liver stiffness, steatosis, fibrosis), as well as metabolic markers (e.g., BMI, glycemic control, insulin resistance, lipid profile)	Studies not reporting MASLD-related or metabolic outcomes, or studies without SCFAs or microbiota data
Study design (S)	Observational studies (cross-sectional, cohort, or case-control) published in peer-reviewed journals	Clinical trials, non-original papers (e.g., reviews, systematic reviews, meta-analyses, clinical trial protocols), case reports, case series, posters, theses, editorials, commentaries; non-English papers; studies that performed interventions at baseline

### Selection of Studies

**Fig. 1:**
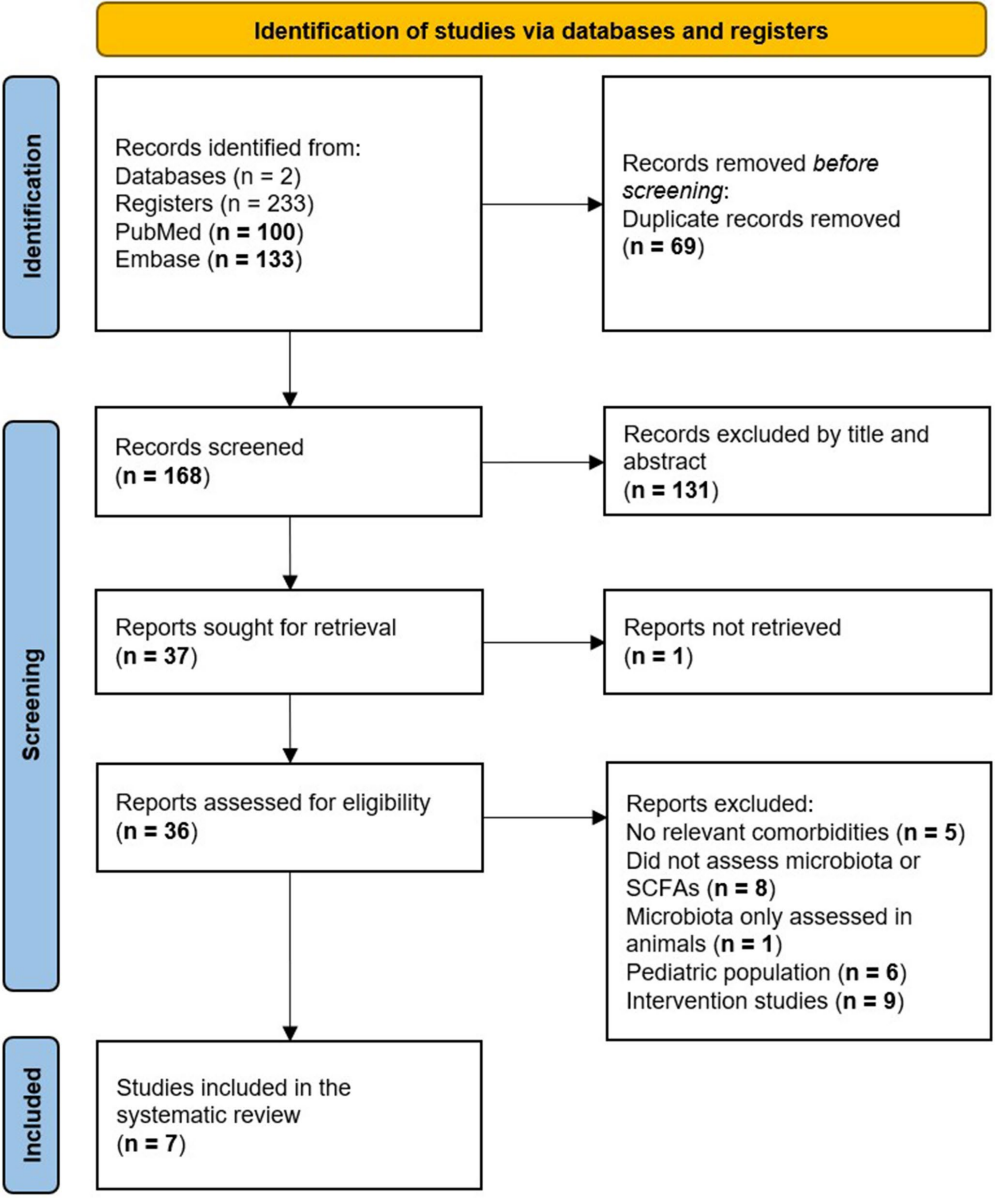
PRISMA flow diagram of study screening and selection

Studies were included based on a set of inclusion and exclusion criteria and relied on the PRISMA flow chart (Fig. 1).

### Data Extraction

Data from the included studies were independently extracted by two authors (VS-U and AG-G) using a standardized data extraction form. The extracted information included study characteristics (author, year, country), study design (cross-sectional, cohort, case-control), population details (sample size, diagnostic criteria for MASLD), sample type for SCFA measurement (feces, plasma or serum), reported abundance of putative SCFA-producing microbiota (taxa reported), metabolic and hepatic outcomes (e.g., BMI, insulin resistance, liver enzymes), and main findings.

Disagreements between authors were resolved by discussion until consensus was reached. If necessary, a third author (JCF-G) was consulted to adjudicate unresolved discrepancies.

### Risk of Bias Assessment

To evaluate the risk of bias of the individual studies included in this systematic review, the methodological quality was assessed using the Newcastle-Ottawa Scale (NOS) by two independent authors (VS-U and AG-G). Any disagreements regarding the scoring were resolved through discussion and, if necessary, adjudicated by a third author (JCF-G).

The NOS is a widely used tool for assessing the quality of non-randomized studies in systematic reviews and meta-analyses. It evaluates study quality based on eight items grouped into three domains: [1] selection of study groups [2], comparability of groups (up to two stars), and [3] ascertainment of either the exposure or the outcome of interest for case-control or cohort studies, respectively. Each study received a score ranging from 0 to 9 stars, with higher scores indicating better methodological quality and a lower risk of bias.

### Data Synthesis and Analysis

Due to the heterogeneity in study designs, populations, outcome measures, and assessment methods across the included studies, a meta-analysis was not feasible. Instead, a qualitative synthesis of the data was performed. Extracted findings were summarized narratively and organized according to key outcome domains, including differences in SCFA levels (especially butyrate), presence or severity of MASLD, and associated metabolic parameters such as insulin resistance, obesity, and lipid profile. Comparisons were drawn between MASLD and control groups when applicable, highlighting consistent patterns or discrepancies. Where relevant, trends were noted regarding the abundance of SCFA-producing microbiota and their associations with metabolic or hepatic markers. Tables were used to present study characteristics and main outcomes for clarity and comparison. Given the observational design of the included studies, findings were interpreted as associative rather than causal.

## Results

### Study Selection

Our initial search yielded 233 records from PubMed and Embase up to April 7, 2025. After removing duplicates, 168 articles remained for title and abstract screening. Of these, 131 were excluded for not meeting the inclusion criteria. Thirty-six full-text articles were assessed for eligibility, resulting in seven studies that met all inclusion criteria and were included in the final analysis (Fig. 1). All selected studies were observational and evaluated associations between SCFA levels or the abundance of SCFA-producing microbiota and MASLD or its related metabolic comorbidities.

### Characteristics of the Included Studies

The seven studies included in this review were published between 2020 and 2025 and involved a total of 1,185 adult participants. These studies were conducted across multiple countries, including Spain, the United Kingdom, Japan, China, and Chile. All studies employed observational designs, either cross-sectional or case-control.

Participants were adults diagnosed with MASLD confirmed by liver biopsy or imaging techniques (e.g., ultrasound or transient elastography), after excluding other known causes of hepatic steatosis (significant alcohol consumption, viral hepatitis, autoimmune liver disease, or drug-induced liver injury). All participants presented at least one associated metabolic comorbidity, such as obesity, insulin resistance, T2D, or increased cardiovascular risk.

**Fig. 2:**
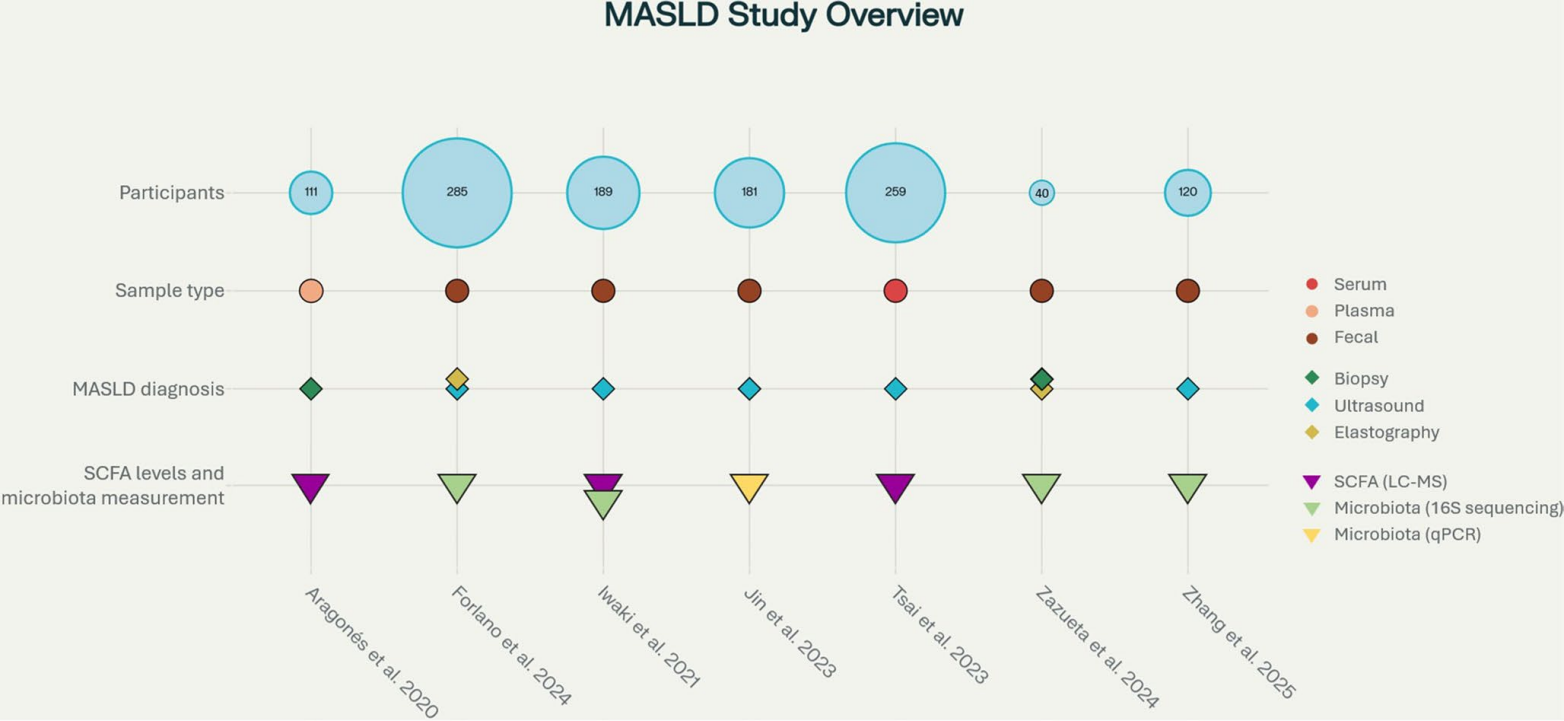
Overview of the main characteristics and methodologies of the included MASLD studies. The graphic summarizes sample size, type of biological sample analyzed (serum, plasma, or fecal), diagnostic methods used for MASLD confirmation (biopsy, ultrasound, or elastography), and the analytical methodologies applied for SCFA quantification and gut microbiota profiling (LC-MS, 16 S rRNA sequencing, or qPCR). Each column represents a study

SCFA concentrations were determined in fecal or plasma samples using liquid chromatography–mass spectrometry, while gut microbiota composition was predominantly assessed through 16 S rRNA gene sequencing in all included studies, except for Jin et al. 2023 [22], who analyzed fecal microbiota by quantitative PCR (qPCR) targeting specific SCFA-producing taxa. Figure 2 provides a visual overview of the design, population characteristics, sample types, diagnostic methods, and main analytical approaches employed in each included MASLD study. Table 3 summarizes the main characteristics of the included studies.

**Table 3:** Main characteristics of the studies included^1^

Reference; country	Sample size	Mean age (years)	Sex (men, %)	Butyrate and other SCFAs, and related microbiota	Sample type	Diagnostic methods for MASLD	MASLD-related parameters	Diabetes-related parameters	Obesity-related parameters	Lipid profile parameters
Aragonès et al. [25]; Spain	**111**: − 29 NW − 82 MO: • 29: MO and NL • 32: MO and SS • 21: MO and MASH	- NW: 41.99 ± 9.20 - MO: 46.31 ± 10.78 • NL: 43.05 ± 10.35 • SS: 47.49 ± 11.54 • MASH: 48.99 ± 9.45	100% women	↓ isobutyrate in MO vs. NW (0.33 vs. 0.47 µM; *p* < 0.001) ↑ isovalerate in MO vs. NW (1.37 vs. 0.25 µM; *p* < 0.001) No sig. differences in SCFA (acetate, propionate, isobutyrate, butyrate, isovalerate) within MO subgroups	Plasma	Biopsy	↑ AST, ALT and GGT in MO vs. NW. No sig. differences within MO subgroups	↑ Glucose, insulin, HOMA-IR, HbA1c in MO vs. NW ↑ Glucose in SS vs. NL; however, in the SS group, there were 10 patients with T2D, and in the MASH group, only 3 No sig. differences in insulin, HOMA-IR and HbA1c within MO subgroups	↑ body weight and BMI in MO vs. NW. No sig. differences within MO subgroups	↑ HDL-C and ↓ TG in NW vs. MO ↑ TG in MASH vs. NL. No sig. differences in TC, LDL-C or HDL-C within MO subgroups
Forlano et al. [27]; United Kingdom	**285**: − 182 T2D and MASLD − 73 T2D and HC	- MASLD: 60 (54–66) - HC: 59 (53–65)	- MASLD: 56% men - HC: 45% men	- **Putative SCFA-producing bacteria in fibrosis**: ↓ *Butyricicoccus* (butyrate), *Ruminococcus* (butyrate), *Dialister* (succinate, acetate, propionate, butyrate), ↑ *Butyricimonas* (butyrate), *Odoribacter* (butyrate), *Acidaminococcus* (acetate and propionate) - **Putative SCFA-producing bacteria in steatosis (MASLD without fibrosis)** ↑ *Butyricicoccus* (butyrate), *Phascolarctobacterium* (↑ propionate)	Fecal: *n* = 97 (20 no liver disease, 58 MASLD/F-, 19 MASLD/F+)	Transient elastography and ultrasound	↑ALT, AST and GGT in MASLD vs. HC	↑Glucose, HbA1c, insulin, HOMA-IR in MASLD vs. HC ↑ Glucose, HbA1c, insulin, HOMA-IR in MASLD/F + vs. MASLD/F-	↑ BMI in MASLD/F + vs. MASLD/F-	No sig. differences in TC, LDL-C, HDL-C or TG within subgroups
Iwaki et al. [24]; Japan	**189**: − 51 MASLD without obesity − 51 MASLD with obesity − 87 HC	- MASLD without obesity: 61.9 ± 14.3 - MASLD with obesity: 57.4 ± 13.3 - HC: 55.6 ± 16.3	- MASLD without obesity: 51% - MASLD with obesity: 47% - HC: 55%	↓ butyrate levels in the MASLD without obesity group vs. MASLD with obesity and HC groups No sig. differences for acetate, propionate, isobutyrate, isovalerate **Putative SCFA-producing microbiota**: ↓*Eubacterium* (butyrate) in the MASLD without obesity group than in the MASLD with obesity and HC groups and correlates with fibrosis severity (*p* = 0.008). ↓ *Faecalibacterium* and *Subdoligranulum* (butyrate) in both MASLD groups vs. HC ↓ *Ruminococcus* (putative SCFA producer) in MASLD without obesity vs. HC	Fecal: *n* = 112 (31 MASLD without obesity, 37 MASLD with obesity and 44 HC)	Ultrasound	↓ ALT in MASLD without obesity vs. MASLD with obesity ↑ALT, AST, GGT in MASLD without obesity vs. HC ↑ AST and GGT in MASLD with obesity vs. HC No sig. differences in AST, GGT, MASLD activity score and pathological findings between MASLD groups	↑ insulin in MASLD without obesity vs. HC	↓ BMI in MASLD without obesity vs. MASLD with obesity, but ↑ BMI in MASLD without obesity vs. HC	Not specified
Jin et al. [22]; China	**181**: − 103 obesity/MASLD − 78 obesity without MASLD	- Obesity/MASLD: 50.98 ± 1.67 - Obesity without MASLD: 55.42 ± 1.92	- Obesity/MASLD: 66% - Obesity without MASLD: 62.8%	↓ *Faecalibacterium prausnitzii* (putative butyrate producer) in obesity/MASLD vs. without MASLD (OR = 0.618, *p* = 0.010) and linked with fibrosis and age-related changes. *F. prausnitzii* positively correlated with other putative SCFA-producing taxa (*Bacteroides*, *Clostridium leptum*, *Clostridium butyricum*, *Eubacterium rectale*) and negatively correlated with *Enterobacteriaceae* VLDL-C was negatively correlated with *F. prausnitzii*	Fecal	Ultrasound	↑ AST, ALT, GGT in obesity/MASLD vs. obesity without MASLD	Tendency to ↑ HbA1c in obesity/MASLD vs. obesity without MASLD. No sig. differences in glucose	↑ BMI in obesity/MASLD vs. obesity without MASLD	Tendency to ↑ TC in obesity/MASLD vs. obesity without MASLD. No sig. differences in LDL-C, HDL-C or TG
Tsai et al. [23]; China	**259**: - 117 T2D with moderate to severe MASLD - 142 T2D without MASLD or with mild MASLD	- Moderate/severe MASLD: 58.8 ± 9.5 - No/mild MASLD: 63.6 ± 10.9	- Moderate/severe MASLD: 59.2% - No/mild MASLD: 57.3%	↓ isobutyrate and ↓ methylbutyrate in moderate/severe MASLD (6.6 vs. 8.6 µM, *p* = 0.003; 5.6 vs. 9.2 µM, *p* = 0.001) and ↑ isovalerate (18.9 vs. 8.1 µM, *p* = 0.04); no differences in acetate, propionate, butyrate, valerate and methylvalerate	Serum	Ultrasound	↑ AST, ALT in moderate/severe MASLD	↓ T2D duration in moderate/severe MASLD ↑ HbA1c in moderate/severe MASLD	↑ BMI in moderate/severe MASLD	↑ TG in moderate/severe MASLD. No sig. differences in TC, LDL-C, or HDL-C
Zazueta et al. [26]; Chile	**40**: − 7 HC − 13 Ow/Ob − 11 MASLD/F− − 9 MASLD/F+	- HC: 39.9 (23–63) - Ow/Ob: 48.9 (18–73) - MASLD/F−: 49.0 (25–67) - MASLD/F+: 63.2 (47–73)	- HC: 71.4% - Ow/Ob: 61.5% - MASLD/F−: 81.8% - MASLD/F+: 33.3%	↓ *Lachnospiraceae* ND3007 and *Defluviitaleaceae* (putative SCFA-producing taxa) in MASLD. ↑ *Ruminococcaceae* UCG-013 (putative butyrate-producing taxon) in MASLD/F+ ↑ *Bifidobacterium* (acetate), *Prevotella* (propionate), *Acidaminococcus* (acetate) positively correlated with liver stiffness and fibrosis	Fecal	Transient elastography and/or histology	↑ AST and ALT in MASLD/F − vs. HC. ↑ GGT in MASLD/F + vs. HC and vs. Ow/Ob	Not specified	↑ weight in Ow/Ob vs. HC and in MASLD/F + vs. HC ↑ BMI in Ow/Ob vs. HC, in MASLD/F- vs. HC, and in MASLD/F + vs. HC	↑ TC in MASLD/F − vs. MASLD/F+ No sig. differences in TG
Zhang et al. [28]; China	**120 participants with overweight/obesity**,** T2D and metabolic dysfunction**: − 60 MASLD patients − 60 HC	**MASLD**: 54.0 (47.25–57.75) **HC**: 52.0 (47.0–57.0)	**MASLD**: 51,6% **HC**: 33.3%	↑ *Eubacterium rectale* (putative butyrate- and propionate-producing taxon) in MASLD; abundance positively associated with propionate levels and ALT. ↑*Phascolarctobacterium* (acetate and propionate), *Lachnospira* (acetate and butyrate) in MASLD; *Lachnospira* abundance negatively associated with ALT and GGT ↓ *Dialister invisus* (succinate, acetate, propionate, butyrate), *Pseudoruminococcus massiliensis*,* Ruminococcaceae spp*., *Clostridiales Family XIII*, *Bifidobacterium pseudocatenulatum* (putative butyrate-producing taxa) in MASLD Additional associations: *Bifidobacterium pseudocatenulatum* abundance negatively associated with HDL-C. *Dialister invisus* abundance negatively associated with ALT *Pseudoruminococcus massiliensis* abundance positively associated with HDL-C. *Ruminococcaceae spp.* abundance negatively associated with TG levels	Fecal	Ultrasound	↑ ALT and GGT in MASLD. No sig. differences in AST	↑ glucose in MASLD	↑ weight and BMI in MASLD	↑ TG, LDL-C in MASLD. ↓ HDL-C in MASLD. No sig. differences in TC

^1^ Cross-sectional and case–control observational studies included in the systematic review

ALT: alanine aminotransferase; AST: aspartate aminotransferase; BMI: body mass index; GGT: gamma-glutamyl transferase; HC: healthy controls; HbA1c: hemoglobin A1c; HDL-C: high-density lipoprotein cholesterol; HOMA-IR: homeostatic model assessment for insulin resistance; LDL-C: low-density lipoprotein cholesterol; MASH: metabolic dysfunction-associated steatohepatitis; MASLD: metabolic dysfunction-associated steatotic liver disease; MASLD/F−: MASLD without fibrosis; MASLD/F+: MASLD with fibrosis; MO: morbid obesity; NL: normal liver; NW: normal weight; Ob: obesity; Ow: overweight; SCFA: short-chain fatty acids; SS: simple steatosis; T2D: type 2 diabetes; TC: total cholesterol; TG: triglycerides; VLDL-C: very low-density lipoprotein cholesterol

Taxa are reported as putative SCFA- or butyrate-producing bacteria based on taxonomic assignment; functional activity was not directly assessed in most studies

### Quality Assessment of the Included Studies

All studies achieved scores ranging from 6 to 8 stars in the Newcastle-Ottawa Scale, indicating a generally moderate to high methodological quality (Table 4). Most studies had well-defined populations and appropriate control groups. Limitations were primarily related to the representativeness of the cohorts and incomplete control for confounders.

**Table 4:** Quality assessment of included studies using the Newcastle-Ottawa scale (case-control studies)

Study	Selection^a^				Comparability^b^		Exposure^c^			Score
	1	2	3	4			A	B	C	
Aragonès et al. [25]	☆	☆	☆	-	☆		☆	☆	-	6
Forlano et al. [27]	☆	☆	☆	☆	☆		☆	☆	-	7
Iwaki et al. [24]	☆	☆	☆	☆	☆		☆	☆	-	7
Jin et al. [22]	☆	☆	-	☆	☆	☆	☆	☆	-	7
Tsai et al. [23]	☆	☆	☆	☆		☆	☆	☆	-	7
Zazueta et al. [26]	☆	☆	☆	☆			☆	☆	-	6
Zhang et al. [28]	☆	☆	☆	☆	☆	☆	☆	☆	-	8

^a^ 1: Is the case definition adequate? 2: Representativeness of the cases. 3: Selection of controls. 4: Definition of controls

^b^ Comparability of cases and controls on the basis of the design or analysis. A maximum of two stars can be given for comparability

^c^ A: Ascertainment of exposure. B: Same method of ascertainment for cases and controls. C: Nonresponse rate

### Analysis of the Included Studies

#### 1. SCFA levels and MASLD severity

A recurrent observation across several studies was an association between lower levels of butyrate, and to a lesser extent other SCFAs (acetate and propionate), and the presence or severity of MASLD. For instance, Tsai et al. [23] and Iwaki et al. [24] measured serum and fecal SCFAs, respectively, and reported significantly lower concentrations of butyrate, isobutyrate, and methylbutyrate in individuals with MASLD, particularly in those with advanced liver fibrosis or MASH. Tsai et al. [23] also noted that participants with more severe steatosis had higher levels of circulating isovalerate. Iwaki et al. [24] specifically found that participants with MASLD in the absence of obesity had markedly lower fecal butyrate concentrations, which were inversely correlated with fibrosis severity. In contrast, Aragonès et al. [25] did not observe significant differences in plasma SCFAs according to MASLD presence or severity among women with morbid obesity.

#### 2. SCFA-Producing Microbiota and MASLD

The reduction in the abundance of different SCFA-producing bacteria emerged as a central finding across the studies. Several studies (e.g., Jin et al. [22], Iwaki et al. [24], Zazueta et al. [26]) described a significant decrease in the relative abundance of key butyrate-producing genera such as *Eubacterium*, *Subdoligranulum*, and *Ruminococcus*, and species such as *Faecalibacterium prausnitzii*, in participants with MASLD compared to healthy controls. These changes were often associated with clinical markers of liver damage and inflammation. In contrast, some studies [e.g., Forlano et al. [27], Zhang et al. [28] ] reported an increased abundance of SCFA-producing genera like *Butyricimonas*, *Odoribacter*, and *Phascolarctobacterium* in individuals with MASLD.

In terms of liver fibrosis, Forlano et al. [27], observed that several SCFA-producing genera - such as *Butyricicoccus* and *Ruminococcus* (butyrate producers), as well as *Dialister* (producer of succinate, acetate, propionate, and butyrate) - were significantly reduced in individuals with MASLD and advanced fibrosis, as assessed by transient elastography. Conversely, genera such as *Butyricimonas* and *Odoribacter* (butyrate producers), and *Acidaminococcus* (producer of acetate and propionate) were found to be increased in this subgroup. Interestingly, in cases of steatosis without fibrosis, an increase in *Butyricicoccus* and *Phascolarctobacterium* (propionate producer) was noted. Furthermore, Zazueta et al. [26] identified a positive correlation between *Ruminococcaceae UCG-013* and liver stiffness, suggesting a potential association between specific microbial taxa and fibrosis severity in MASLD patients.

#### 3. Liver Parameters

Individuals with MASLD exhibited elevated levels of liver enzymes (alanine aminotransferase [ALT], aspartate aminotransferase [AST], gamma-glutamyl transferase [GGT]) in the studies included in the systematic review.

Zhang et al. [28] provided further insight through correlation analyses. *Eubacterium rectale* (butyrate and propionate producer) was increased in MASLD and positively associated with propionic acid levels and ALT. Additionally, *Phascolarctobacterium* (acetate and propionate producer) and *Lachnospira* (acetate and butyrate producer) were also elevated in MASLD; *Lachnospira* showed a negative correlation with ALT and GGT. Conversely, *Dialister invisus* (butyrate producer) was reduced in MASLD, and negatively associated with ALT.

#### 4. Metabolic Parameters

People with MASLD showed increased insulin resistance (as indicated by the homeostatic model assessment for insulin resistance [HOMA-IR], fasting insulin, and glucose), and higher BMI or body weight compared to healthy or non-MASLD controls.

Furthermore, regarding BMI, Iwaki et al. [24] reported a reduction in *Eubacterium* (butyrate-producing) in MASLD without obesity, compared to both MASLD with obesity and to controls. Its abundance inversely correlated with fibrosis severity assessed by serum fibrosis markers and liver biopsy. Jin et al. [22] also observed decreased abundance of *Faecalibacterium prausnitzii* (a major butyrate producer) in people with MASLD and obesity compared to those with simple obesity, with associations to fibrosis and age-related changes. Zazueta et al. [26]. reported reduced *Bifidobacterium* (acetate producer), *Prevotella* (propionate producer), and *Acidaminococcus* (acetate producer) in MASLD, with positive correlations between these taxa and liver stiffness or fibrosis severity.

Additionally, alterations in lipid profile were commonly described, although results varied across studies. In line with this, Aragonès et al. [25], Tsai et al. [23] and Zhang et al. [28] reported higher triglyceride levels and/or lower high-density lipoprotein cholesterol (HDL-C) in patients with MASLD compared to those without MASLD. Jin et al. [22] and Zazueta et al. [26] also observed elevated total cholesterol levels in these individuals. However, only a few studies directly evaluated whether these dyslipidemic changes were associated with SCFAs levels or gut microbiota composition. For instance, Jin et al. [22] found that *Faecalibacterium prausnitzii* was inversely associated with very low-density lipoprotein cholesterol (VLDL-C) levels and positively correlated with other SCFA-producing genera. In addition, Zhang et al. [28] reported that *Bifidobacterium pseudocatenulatum* (butyrate producer) were reduced in MASLD, and negatively correlated with HDL-C. *Pseudoruminococcus massiliensis* (also reduced in MASLD) was positively associated with HDL-C, whereas *Ruminococcaceae* spp. were negatively correlated with triglyceride levels, further supporting links between gut microbiota composition, SCFA profiles, and lipid homeostasis in MASLD.

#### 5. Evidence Map of Associations

**Fig. 3:**
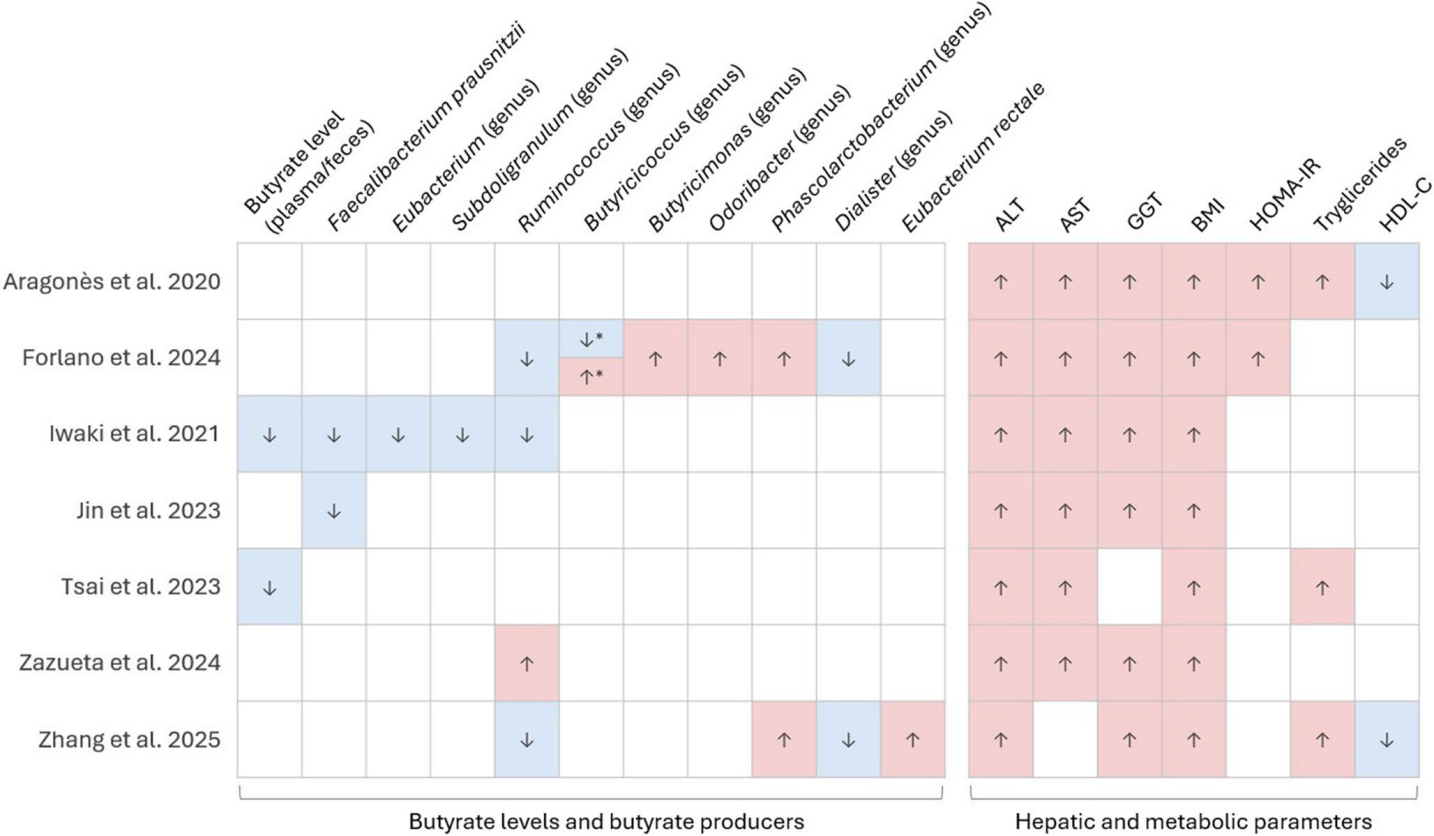
Evidence map (heatmap) showing associations between butyrate-producing taxa, SCFAs, and hepatic or metabolic parameters across included studies. Colors and arrows of each cell denote direction of association: red (up arrow) for positive, blue (down arrow) for negative, and white (no arrow) for no association. * Stratified analyses yield both positive and negative associations for the same taxon: negative association in MASLD with fibrosis, and positive association in MASLD without fibrosis

To provide a comprehensive overview of the relationships between butyrate-producing bacteria, SCFA profiles, and hepatic and metabolic parameters in adults with MASLD, we generated an evidence map in the form of a heatmap (Fig. 3). In this visualization, the direction of each reported association is standardized as follows: red and up arrow indicates a positive association, blue and down arrow denotes a negative association, and white with no arrow indicates no association.

Some studies performed stratified analyses, which may result in both positive and negative associations for the same microbial taxon. For example, in Forlano et al., certain butyrate-producing genera showed decreased abundance in individuals with fibrosis, while others were increased, leading to mixed signals within the same study [27].

The bottom section of the heatmap summarizes the overall patterns observed for butyrate and butyrate-producing taxa, allowing rapid comparison across the included studies.

## Discussion

This systematic review synthesizes current evidence regarding the role of SCFAs - particularly butyrate - and butyrate-producing gut bacteria in the presence and severity of MASLD and its metabolic comorbidities. By focusing exclusively on observational human studies, this systematic review avoids the heterogeneity introduced by intervention protocols and provides a coherent overview of reported associations between SCFA levels, or their microbial producers, and the presence or severity of MASLD across diverse populations. Most included studies report alterations in SCFAs levels, particularly butyrate, together with shifts in the abundance of SCFA-producing microbiota in individuals with MASLD and associated metabolic comorbidities. These findings support an association between butyrate-related alterations and MASLD pathophysiology.

Several studies observed decreased levels of butyrate or related SCFAs - such as isobutyrate or methylbutyrate - in patients with MASLD, especially in those with more advanced disease stages or liver fibrosis. These findings support the hypothesis that intestinal dysbiosis, marked by reduced SCFA production, may be involved in MASLD pathophysiology and disease severity. Butyrate depletion is associated with impaired gut barrier function and systemic inflammation, mechanisms thought to play key roles in MASLD pathophysiology and liver injury exacerbation [29]. Furthermore, one study also noticed that participants with more advanced MASLD presented higher levels of isovalerate, suggesting a shift in microbial fermentation patterns as disease severity increases.

An important conceptual consideration when interpreting these findings relates to causality. Although reduced butyrate levels and alterations in butyrate-producing microbiota are consistently associated with greater MASLD severity, the observational nature of all included studies precludes causal inference. It therefore remains unclear whether reduced butyrate availability represents a causal driver of disease progression, a downstream consequence of metabolic dysfunction and dietary patterns, or a biomarker reflecting overall metabolic status. Accordingly, the associations described in this review should be interpreted as correlational rather than causal.

In addition, it is important to distinguish between related but biologically distinct layers frequently reported across studies, including measured fecal or circulating SCFA concentrations, the taxonomic abundance of putative butyrate-producing bacteria, and the inferred microbial functional capacity for SCFA production. These layers are not interchangeable and may capture different aspects of host–microbiota interactions. Consequently, mechanistic interpretations should be made with caution, particularly when functional inferences are based solely on taxonomic data.

**Fig. 4:**
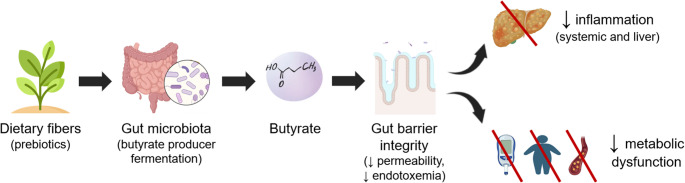
Schematic summary of the gut–liver axis in MASLD. Dietary fibers are fermented by butyrate-producing bacteria into butyrate, which improves gut barrier integrity (reducing permeability and endotoxemia), thereby attenuating systemic and liver inflammation and metabolic dysfunction

Figure 4 summarizes the core biological pathways proposed to link altered SCFA-producing microbiota with MASLD progression. The schematic integrates current evidence suggesting that reduced abundance of butyrate-producing bacteria may be associated with lower butyrate availability, impaired intestinal barrier integrity, and increased translocation of pro-inflammatory mediators. These mechanisms have been proposed to contribute to hepatic steatosis, inflammation, and fibrosis, while simultaneously contributing to systemic metabolic dysfunction—including insulin resistance and dyslipidemia—frequently observed in MASLD. Incorporating these mechanisms into a unified framework helps contextualize the heterogeneous SCFA and microbiota alterations reported across studies and supports a gut–liver axis–based model for MASLD pathophysiology.

Parallel alterations are evident in the composition of butyrate-producing microbiota. Consistent with reduced butyrate levels, reductions in key butyrate-producing genera such as *Eubacterium*, *Subdoligranulum*, and *Ruminococcus*, and species such as *Faecalibacterium prausnitzii*, are observed across several studies. These findings support the notion that MASLD is characterized by a disruption in the balance of beneficial SCFA-producing microbiota, which may influence hepatic outcomes. However, it should be noted that some studies have also reported a higher abundance of butyrate producers in MASLD, or even showing an increase in some butyrate producers with decrease of others, reflecting the complexity of these associations with MASLD and suggesting that the metabolic effects of butyrate and other SCFAs may be context-dependent. Furthermore, with respect to fibrosis, whereas some studies have reported decreased abundances of various butyrate-producing bacteria, others have identified increases in certain genera, highlighting the complexity of microbial alterations, possibly suggesting the presence of stage-dependent shifts within the SCFA-producing microbiota, as previously suggested [30].

Importantly, evidence from obesity and metabolic disease research suggests that the metabolic effects of butyrate and other SCFAs may not be uniformly beneficial, but rather highly context-dependent. In individuals with obesity, increased SCFA production has been associated with enhanced energy harvest efficiency and greater caloric availability from the diet, potentially contributing to weight maintenance or weight gain in susceptible metabolic contexts [31]. Conversely, in other settings—such as insulin resistance, low-grade inflammation, or during weight-loss interventions—butyrate has been linked to improved metabolic flexibility, enhanced insulin sensitivity, and reduced inflammatory tone, as supported by mechanistic and preclinical evidence [32]. These observations indicate that the physiological impact of butyrate depends on host metabolic status, dietary patterns, and disease stage, and should therefore be interpreted cautiously when extrapolating its role across different metabolic conditions.

Nevertheless, these discrepancies likely also reflect heterogeneity among studies. First, differences in sample types (e.g., fecal versus plasma SCFA measurements) may have influenced the detected SCFA concentrations. Plasma levels reflect both intestinal production and systemic absorption, while fecal levels represent luminal content, influenced by dietary carbohydrates, presence of growth factors and oxygen concentrations [33]. Furthermore, some of the studies included in the systematic review do not evaluate SCFAs directly, but rather SCFA-producing bacteria. Such methodological variability may obscure true associations with hepatic or metabolic outcomes.

Second, sex distribution also emerges as a potential confounder. Aragonès et al. [25] included only female participants, while most other studies included both sexes. Given known sex differences in gut microbiota composition, SCFA metabolism, and MASLD prevalence or risk of associated complications [34, 35], these demographic imbalances may partially explain the inconsistent microbial findings.

Third, population metabolic profiles varied notably. While some studies focused on individuals with obesity or T2D, others included people with MASLD without obesity or metabolically heterogeneous MASLD populations. In this sense, microbial composition and SCFA production are strongly influenced by host metabolic state [36]. For instance, Iwaki et al. [24] highlighted specific alterations in butyrate-producing bacteria in participants with MASLD without obesity that may differ or be absent in populations with MASLD and obesity. T2D, as a condition characterized by metabolic dysfunction, also exerts an influence on gut microbiota composition. Thus, Tsai et al. [23] found differences regarding serum SCFA levels in people with T2D, depending on MASLD stage (moderate/severe compared to no/mild MASLD), whilst Forlano et al. [27] reported distinct alterations in SCFA-producing bacteria in people with T2D, depending on the stage of MASLD progression (with or without fibrosis).

Geographic and dietary factors may also contribute to variability. Studies from Asia (e.g., Japan, China) versus Europe or South America (e.g., Spain, Chile) involve populations with distinct dietary habits that strongly affect microbiota composition and SCFA production. Fiber intake and consumption of fermented foods vary widely across cultures and may modulate SCFA profiles independently of liver disease status [37].

Importantly, dietary intake represents a central upstream determinant of both gut microbiota composition and SCFA production. Variations in fiber quantity and type, plant-based food consumption, and intake of ultra-processed foods may strongly influence butyrate availability, independently of liver disease status [37]. In this context, reduced butyrate levels observed in MASLD populations may partly reflect poorer overall dietary quality rather than disease-specific microbial dysfunction, reinforcing the need to interpret microbiota–butyrate associations within a broader dietary and metabolic framework.

Taken together, these methodological and biological sources of heterogeneity underscore the complexity of interpreting SCFA-related microbiota changes in MASLD. Rather than contradictory, the observed discrepancies may reflect distinct microbiota signatures associated with different MASLD phenotypes across populations and disease stages, since the metabolic effects of butyrate may be context-dependent, particularly in relation to host metabolic state. Future research should stratify analyses by sex, obesity status, geographic region, and dietary intake, and ideally adopt longitudinal designs to clarify whether microbiota alterations precede or follow MASLD development and progression.

On the other hand, MASLD was generally associated with elevated hepatic enzymes (ALT, AST, GGT), increased markers of insulin resistance (e.g., HOMA-IR, insulin, glucose, HbA1c), and obesity-related indicators (e.g., BMI, body weight), reflecting the metabolic disturbances central to MASLD pathophysiology. Several included studies suggest these clinical markers may be modulated by gut microbiota composition and SCFA levels, particularly butyrate. For instance, reduced abundance of *Faecalibacterium prausnitzii* and other butyrate producers has been linked to increased ALT, VLDL-C, and systemic inflammation markers, reinforcing the hypothesis that butyrate depletion may be associated with hepatic and metabolic dysfunction, as well as inflammation.

Moreover, Zhang et al. [28] reported that reduced abundance of other butyrate producers was also linked to changes in liver profile. Thus, *Dialister invisus* was inversely associated with ALT, while taxa such as *Pseudoruminococcus massiliensis* and *Ruminococcaceae spp.* correlated positively with HDL-C and negatively with triglyceride levels, respectively. These findings highlight a potential role for butyrate producers in modulating liver injury and lipid metabolism. Notably, Jin et al. [22]. found that *Faecalibacterium prausnitzii* was inversely associated with VLDL-C and positively correlated with other butyrate producers, indicating a protective role in lipid metabolism and liver health. The role of other SCFA-producing bacteria, however, remains unclear. For instance, *Lachnospira* —an acetate and butyrate producer— showed a negative correlation with ALT and GGT, but also both low and elevated levels in MASLD have been reported [26, 28]. However, it should be noted that these associations were predominantly observed for butyrate-producing bacteria, and further investigation is needed to evaluate the correlation between these outcomes and fecal or circulating butyrate concentrations. Interestingly, associations between butyrate and metabolic parameters exist in populations without MASLD. In individuals with T2D, lower fecal or circulating butyrate levels associate with poorer glycemic control, higher insulin resistance, and adverse lipid profile [38]. This suggests that SCFA dysregulation in MASLD may reflect a broader metabolic phenotype wherein impaired SCFA production contributes to systemic metabolic dysfunction.

Furthermore, several studies have demonstrated that metabolic improvements after bariatric surgery are accompanied by taxonomical and functional shifts in the gut microbiota, including an increased abundance of butyrate-producing bacteria such as *Faecalibacterium prausnitzii*, suggesting a potential role for butyrate in MASLD regression/resolution [39].

Overall, these results indicate that the link between SCFA-producing microbiota and metabolic markers - such as insulin resistance and lipid abnormalities - may not be exclusive to MASLD, but rather part of a common mechanistic pathway in metabolic diseases. However, liver involvement in MASLD may potentiate or modify these associations, underscoring the need for further observational and mechanistic studies, both to clarify the links between microbial metabolites - especially butyrate - and MASLD progression, and to elucidate the interaction between MASLD, metabolic comorbidities, and microbiota. Nevertheless, the combined depletion of fecal butyrate and key producers such as *Faecalibacterium prausnitzii* may represent a non-invasive biomarker for MASLD severity and may help stratify patients for dietary or microbiota-directed therapies.

Our systematic review has certain limitations but also some important strengths. The limitations include the observational design of the included studies, where only an association and not a cause can be inferred. Additionally, the number of eligible studies was limited, and there was substantial heterogeneity in study design, SCFA quantification techniques, microbial profiling methods, and participant characteristics. A key methodological limitation of the included studies is the predominant reliance on 16 S rRNA gene sequencing to characterize gut microbiota composition. Although this approach provides valuable taxonomic information, it lacks the resolution required to accurately identify butyrate-producing microorganisms at the species, subspecies, or strain level. Moreover, 16 S rRNA sequencing does not allow direct assessment of functional metabolic pathways involved in SCFA production, nor does it capture microbial gene expression or activity. As a result, inferences regarding butyrate production are often indirect and based on taxonomic proxies rather than functional evidence. In addition, none of the included studies assessed the gut virome or bacteriophages, which may modulate bacterial ecology and metabolic output. Future studies integrating shotgun metagenomics, metatranscriptomics, metabolomics, and functional pathway analyses will be essential to more precisely define the role of butyrate-producing microbes and their metabolic activity in MASLD. The presence of comorbidities was also variably reported or controlled, potentially influencing microbiota and SCFAs levels. Moreover, included studies did not adjust for dietary intake or the use of medications (especially glucagon-like peptide-1 receptor agonists, sodium-glucose cotransporter 2 inhibitors, or statins). Notably, some studies did not measure SCFAs levels directly but inferred microbial function from taxonomic abundance, which could introduce misclassification bias. Another limitation is the MASLD diagnosis; while liver biopsy is the gold-standard, most studies used liver ultrasound (which can detect liver steatosis but not MASH/liver fibrosis) or transient elastography (which may lack sufficient sensitivity to stage fibrosis accurately or diagnose MASH). Consequently, some patients may have been misclassified regarding disease stage.

Despite these limitations, our systematic review also has several notable strengths. Firstly, it follows a rigorous and reproducible methodology, including systematic searches of two major databases, clearly defined PICOS criteria, duplicate independent screening and data extraction by two reviewers, and independent quality assessment using the Newcastle-Ottawa Scale. In line with this, we pre-registered our protocol in PROSPERO in order to enhance the transparency and methodological integrity of our research. Furthermore, all included studies were rated as moderate to high quality, supporting the reliability of the findings. Besides, the overall sample of the studies included in our systematic review was large, surpassing 1,000 participants, which contributes to enhance the robustness of the findings observed. Additionally, by focusing exclusively on observational human studies from diverse international cohorts, this review avoids confounding from interventional designs and provides a comprehensive synthesis of real-world associations between butyrate levels, butyrate-producing microbiota, MASLD severity, and metabolic comorbidities. Importantly, this review addresses a relatively unexplored topic - the role of butyrate and butyrate-producing gut bacteria in MASLD and its metabolic comorbidities - providing novel insights into the gut–liver axis and SCFA metabolism in MASLD pathophysiology. Finally, these insights offer actionable implications for future research, including the potential of butyrate-related biomarkers for MASLD risk stratification and the prioritization of dietary or microbiota-targeted interventions in high-risk metabolic populations.

## Conclusion

This systematic review highlights an association between reduced butyrate availability and alterations in butyrate-producing gut taxa with MASLD presence and severity, reinforcing the relevance of the gut–liver axis and SCFA-related mechanisms in disease pathophysiology. While these findings are mechanistically plausible and may represent candidate non-invasive biomarkers for disease stratification, pending validation in prospective studies, the observational nature of the evidence and substantial methodological heterogeneity preclude causal inference. Larger, well-phenotyped, multicentre studies using standardized SCFA quantification (including specimen type and analytical platforms), dietary and medication ascertainment, and functional microbiome profiling are needed to validate these signals and clarify their diagnostic and therapeutic implications. Until then, these associations warrant cautious interpretation and further validation through longitudinal and mechanistic studies. 

## Key References

• EASL–EASD–EASO Clinical Practice Guidelines on MASLD. J Hepatol. 2024. — Of outstanding importance.
  - ◦ These multidisciplinary guidelines outline contemporary MASLD diagnosis and management and emphasize the need for robust biomarkers. They also contextualize the role of microbiota-derived metabolites in disease pathophysiology, including SCFAs such as butyrate.
• Forlano R et al. Gut Microbes. 2024. — Of importance.
  - ◦ This observational study links gut barrier dysfunction and microbial alterations with MASLD severity in patients with T2D, supporting biological mechanisms consistent with reduced butyrate production.
• Zazueta N et al. 2024. Int J Mol Sci — Of importance.
  - ◦ This study is the only recent investigation within the systematic review that includes liver biopsy—the gold standard for characterizing MASLD severity. By comparing healthy controls, obesity without MASLD, MASLD without fibrosis, and MASLD with fibrosis, it provides uniquely detailed stratification across disease stages. Despite its modest sample size, the biopsy-based assessment offers strong diagnostic precision and important insight into microbiota–fibrosis associations.

## Supplementary Information

Below is the link to the electronic supplementary material.

Supplementary Material 1

## Data Availability

Data will be available on reasonable request to the corresponding authors.
