# Supplementary material for: The Role of Butyrate in People with Metabolic Dysfunction-Associated Steatotic Liver Disease and Related Metabolic Comorbidities: A Systematic Review
Source: Curr Obes Rep. 2026 Mar 4;15(1):17. doi: 10.1007/s13679-026-00694-8 (PMC12960490; doi:10.1007/s13679-026-00694-8)
Supplement: Supplementary file 1 — Supplementary Material 1 [file 13679_2026_694_MOESM1_ESM.pdf]

NIHR | National Institute for Health and Care Research

PROSPERO
International prospective register of systematic reviews

The Role of Butyrate in People with Metabolic Dysfunction-Associated Steatotic Liver Disease and Related Metabolic Comorbidities: A Systematic Review

Alicia González González, Virginia Soria Utrilla, José Carlos Fernández García

Citation

Alicia González González, Virginia Soria Utrilla, José Carlos Fernández García. The Role of Butyrate in People with Metabolic Dysfunction-Associated Steatotic Liver Disease and Related Metabolic Comorbidities: A Systematic Review. PROSPERO 2025 CRD420251162439. Available from https://www.crd.york.ac.uk/PROSPERO/view/CRD420251162439.

REVIEW TITLE AND BASIC DETAILS

Review title

The Role of Butyrate in People with Metabolic Dysfunction-Associated Steatotic Liver Disease and Related Metabolic Comorbidities: A Systematic Review

Condition or domain being studied

Hepatic Steatosis; Non-alcoholic fatty liver disease; Obesity; Type 2 Diabetes Mellitus; Insulin Resistance; Metabolic Syndrome

Rationale for the review

Metabolic dysfunction-associated steatotic liver disease (MASLD) is the most common chronic liver disorder worldwide and a major cause of metabolic and cardiovascular morbidity. Growing evidence highlights the gut–liver axis as a key modulator of MASLD development and progression, with alterations in gut microbiota composition and microbial metabolites—particularly short-chain fatty acids (SCFAs)—implicated in its pathophysiology. Among SCFAs, butyrate has shown protective effects on gut barrier integrity, inflammation, and hepatic metabolism in preclinical models. However, human data remain inconsistent, with heterogeneous findings regarding butyrate levels and the abundance of butyrate-producing bacteria across MASLD populations.

This systematic review aims to synthesize current observational human evidence linking endogenous butyrate and butyrate-producing gut microbiota with MASLD and its metabolic comorbidities. By focusing exclusively on human observational studies, this review will provide an updated and clinically relevant overview of how alterations in SCFA metabolism and related

microbiota may contribute to MASLD development and progression. The findings may help clarify potential diagnostic or therapeutic roles for butyrate within the gut–liver axis.

Review objectives
The objective of this systematic review is to synthesize current clinical evidence on the associations between endogenous butyrate—either measured directly as a short-chain fatty acid (SCFA) or inferred from the abundance of butyrate-producing gut bacteria—and the presence or severity of metabolic dysfunction-associated steatotic liver disease (MASLD) and its related metabolic comorbidities.

Specifically, the review aims to:

Examine differences in butyrate levels and butyrate-producing microbiota between individuals with MASLD and healthy controls.

Explore associations between butyrate or SCFA-producing bacteria and hepatic outcomes (e.g., liver enzymes, steatosis, fibrosis, or liver stiffness).

Assess relationships between butyrate or SCFA-related microbiota and metabolic parameters commonly altered in MASLD (e.g., insulin resistance, glucose and lipid metabolism, body mass index).

Identify gaps and methodological heterogeneity in existing observational evidence to guide future research on the gut–liver axis in MASLD.

Keywords
Butyrate; Butyrate-producing bacteria; Gut microbiota; Insulin resistance; Liver fibrosis; Metabolic Comorbidities; Metabolic dysfunction-associated steatotic liver disease; Short-chain fatty acids

Country
Spain

ELIGIBILITY CRITERIA

Population

Included
Adults with MASLD and associated metabolic comorbidities (obesity, diabetes, insulin resistance, metabolic syndrome, or cardiovascular risk).

Excluded
Studies conducted in animals or in vitro; studies in pediatric populations.

Intervention(s) or exposure(s)

Included
Butyrate; Short chain fatty acid

Presence of MASLD (any grade).

Excluded
Studies not assessing MASLD.

Comparator(s) or control(s)

Included
Healthy controls without MASLD (or without metabolic comorbidities).

Excluded
Studies lacking a comparator group or using inappropriate comparison groups for MASLD status.

Study design

Only nonrandomized study types will be included.

Included
This review will include observational studies, specifically cross-sectional, case-control, and cohort studies that assess the relationship between short-chain fatty acids (SCFAs), gut microbiota composition, and MASLD or its severity (steatosis, steatohepatitis, or fibrosis). Interventional studies will be included only if they report baseline data on SCFAs or microbiota composition in MASLD participants.

Excluded
Animal studies, in vitro studies, case reports, narrative reviews, editorials, and conference abstracts without full data will be excluded. Randomised clinical trials will also be excluded unless relevant baseline data for MASLD participants are provided.

Context

The review will include studies conducted in clinical and community settings, including hospitals, outpatient clinics, and primary care centers. Both adult and pediatric populations will be considered where relevant. Studies from all geographic regions will be included; however, research focusing exclusively on low- and middle-income countries will be highlighted in subgroup analyses. Settings with specialized interventions, such as bariatric or metabolic clinics, will also be eligible. Studies conducted in non-healthcare environments (e.g., purely laboratory-based or animal studies) will be excluded unless directly relevant to human clinical outcomes.

TIMELINE OF THE REVIEW

Date of first submission to PROSPERO

06 October 2025

Review timeline

Start date: 1 October 2025. End date: 4 November 2025.

Date of registration in PROSPERO

07 October 2025

AVAILABILITY OF FULL PROTOCOL

Availability of full protocol

A full protocol has been written but is not available because:

A full protocol has been written but is not yet publicly available as it is still under internal review

SEARCHING AND SCREENING

Search for unpublished studies

Only published studies will be sought.

Main bibliographic databases that will be searched

The main databases to be searched are *Embase.com* and *PubMed*.

Search language restrictions

The review will only include studies published in English.

Search date restrictions

Databases will be searched for articles published from 1 January 2020 and before by 7 April 2025.

Other methods of identifying studies

No other methods will be used.

Link to search strategy

A full search strategy is available in the full protocol as described in the *Availability of full protocol* section

Selection process

Studies will be screened independently by at least two people (or person/machine combination) with a process to resolve differences.

Other relevant information about searching and screening

None

DATA COLLECTION PROCESS

Data extraction from published articles and reports

Data will be extracted independently by at least two people (or person/machine combination) with a process to resolve differences.

Authors will be asked to provide any required data not available in published reports.

Study risk of bias or quality assessment

Risk of bias will be assessed using: *Newcastle-Ottawa*

Data will be assessed independently by at least two people (or person/machine combination) with a process to resolve differences.

Additional information will be sought from study investigators if required information is unclear or unavailable in the study publications/reports.

Reporting bias assessment

Risk of bias due to missing results will be assessed

Certainty assessment

To evaluate the risk of bias of the individual studies included in this systematic review, the methodological quality was assessed using the Newcastle-Ottawa Scale (NOS) by two independent reviewers (VS-U and AG-G). Any disagreements regarding the scoring were resolved through discussion and, if necessary, adjudicated by a third author (JCF-G).

The NOS is a widely used tool for assessing the quality of non-randomized studies in systematic reviews and meta-analyses. It evaluates study quality based on eight items grouped into three domains: (1) selection of study groups, (2) comparability of groups (up to two stars), and (3) ascertainment of either the exposure or the outcome of interest for case-control or cohort studies, respectively. Each study received a score ranging from 0 to 9 stars, with higher scores indicating better methodological quality and a lower risk of bias.

OUTCOMES TO BE ANALYSED

Main outcomes

Differences in SCFAs levels or SCFAs-producing microbiota, and its relationship with MASLD-related parameters (e.g., liver enzymes, liver stiffness, steatosis, fibrosis), as well as metabolic markers (e.g., BMI, insulin resistance, lipid profile).

Additional outcomes

There are no additional outcomes.

PLANNED DATA SYNTHESIS

Strategy for data synthesis

Due to the heterogeneity in study designs, populations, outcome measures, and assessment methods across the included studies, a meta-analysis was not feasible. Instead, a qualitative synthesis of the data was performed. Extracted findings were summarized narratively and organized according to key outcome domains, including differences in SCFAs levels (especially butyrate), presence or severity of MASLD, and associated metabolic parameters such as insulin resistance, obesity, and lipid profile. Comparisons were drawn between MASLD and control groups when applicable, highlighting consistent patterns or discrepancies. Where relevant, trends were noted regarding the abundance of SCFAs-producing microbiota and their associations with metabolic or hepatic markers. Tables were used to present study characteristics and main outcomes for clarity and comparison.

CURRENT REVIEW STAGE

Stage of the review at this submission

Review stage	Started	Completed
Pilot work	✓	✓
Formal searching/study identification	✓	✓
Screening search results against inclusion criteria	✓	✓

Review stage	Started	Completed
Data extraction or receipt of IPD		
Risk of bias/quality assessment		
Data synthesis		

Review status
The review is currently planned or ongoing.

Publication of review results
Results of the review will be published.

REVIEW AFFILIATION, FUNDING AND PEER REVIEW

Review team members
Dr Alicia González González (review guarantor and contact) ORCID: 0000-0001-8020-2531. IBIMA. Spain.

No conflict of interest declared.

Mrs Virginia Soria Utrilla. Hospital Regional Universitario de Málaga, Málaga. Instituto de Investigación Biomédica de Málaga y Plataforma en Nanomedicina (IBIMA-Plataforma BIONAND), Málaga, Spain.. Spain.

No conflict of interest declared.

Dr José Carlos Fernández García. Hospital Regional Universitario de Málaga, Málaga. Instituto de Investigación Biomédica de Málaga y Plataforma en Nanomedicina (IBIMA-Plataforma BIONAND), Málaga, Spain.. Spain.

No conflict of interest declared.

Named contact
Dr Alicia González González (alicia.gonzalez@ibima.eu). ORCID: 0000-0001-8020-2531. IBIMA. Spain.

Review affiliation
Faculty of Medicine, University of Málaga

Hospital Universitario Virgen de la Victoria, Málaga. IBIMA-Plataforma BIONAND

Funding source
Review has no specific/external funding but is supported by guarantor/review team (non-commercial) institutions.

Peer review
The review protocol has undergone peer review by experts in endocrinology, nutrition, and metabolic diseases, providing feedback on methodology and study selection.

ADDITIONAL INFORMATION

Review conflict of interest

Declared individual interests are recorded under team member details.. No additional interests are recorded for this review.

Medical Subject Headings

Butyrates; Liver Diseases; Microbiota; Fatty Acids, Volatile

SIMILAR REVIEWS

Check for similar records already in PROSPERO

PROSPERO identified a number of existing PROSPERO records that were similar to this one (last check made on 6 October 2025). These are shown below along with the reasons given by that the review team for the reviews being different and/or proceeding.

- Short-Chain Fatty Acids and Colorectal Cancer: Microbiome-Metabolome Interactions and Intervention Efficacy: A Pooling Up Analysis. [published 28 September 2025] [CRD420251157250]. The review was judged **not to be similar**
- Efficacy and Safety of Resmetirom in Treating Patients With Metabolic Dysfunction-associated Steatotic Liver Disease (MASLD): a Systematic Review and Meta-analysis [published 14 February 2024] [CRD42024509494]. The review was judged **not to be similar**
- Metabolic Dysfunction-associated Steatotic Liver Disease (MASLD) vs Alcohol-related Liver Disease (ALD): liver DNA methylation analysis. A systematic review. [published 22 February 2024] [CRD42024511974]. The review was judged **not to be similar**
- Metabolic dysfunction-associated steatotic liver disease (MASLD) and the risk of comorbidities: a comprehensive systematic review and meta-analysis [published 17 December 2024] [CRD42024615726]. The review was judged **not to be similar**

PROSPERO version history

- Version 1.0, published 07 Oct 2025

Disclaimer

The content of this record displays the information provided by the review team. PROSPERO does not peer review registration records or endorse their content.

PROSPERO accepts and posts the information provided in good faith; responsibility for record content rests with the review team. The guarantor for this record has affirmed that the information provided is truthful and that they understand that deliberate provision of inaccurate information may be construed as scientific misconduct.

PROSPERO does not accept any liability for the content provided in this record or for its use. Readers use the information provided in this record at their own risk.

Any enquiries about the record should be referred to the named review contact
